# Stacking Interactions and Flexibility of Human Telomeric Multimers

**DOI:** 10.1021/jacs.3c04810

**Published:** 2023-07-11

**Authors:** Benedetta Petra Rosi, Valeria Libera, Luca Bertini, Andrea Orecchini, Silvia Corezzi, Giorgio Schirò, Petra Pernot, Ralf Biehl, Caterina Petrillo, Lucia Comez, Cristiano De Michele, Alessandro Paciaroni

**Affiliations:** † Department of Physics and Geology, University of Perugia, Via Alessandro Pascoli, 06123 Perugia, Italy; ‡ CNR-IOM, Department of Physics and Geology, University of Perugia, Via Alessandro Pascoli, 06123 Perugia, Italy; § CNRS, Institut de Biologie Structurale, 71 Avenue des Martyrs, 38044 Grenoble, France; ∥ European Synchrotron Radiation Facility (ESRF), 71 Avenue des Martyrs, 38043 Grenoble, France; ⊥ Jülich Centre for Neutron Science and Institute of Biological Information Processing (JCNS-1/IBI-8), Forschungszentrum Jülich GmbH, 52425 Jülich, Germany; # Department of Physics, University of Rome La Sapienza, Piazzale Aldo Moro 2, 00185 Rome, Italy

## Abstract

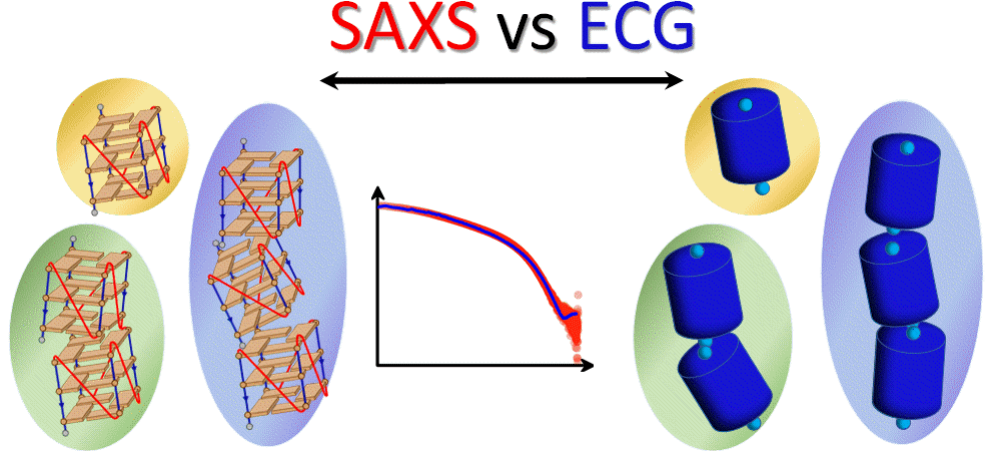

G-quadruplexes (G4s) are helical four-stranded structures forming from guanine-rich nucleic acid sequences, which are thought to play a role in cancer development and malignant transformation. Most current studies focus on G4 monomers, yet under suitable and biologically relevant conditions, G4s undergo multimerization. Here, we investigate the stacking interactions and structural features of telomeric G4 multimers by means of a novel low-resolution structural approach that combines small-angle X-ray scattering (SAXS) with extremely coarse-grained (ECG) simulations. The degree of multimerization and the strength of the stacking interaction are quantitatively determined in G4 self-assembled multimers. We show that self-assembly induces a significant polydispersity of the G4 multimers with an exponential distribution of contour lengths, consistent with a step-growth polymerization. On increasing DNA concentration, the strength of the stacking interaction between G4 monomers increases, as well as the average number of units in the aggregates. We utilized the same approach to explore the conformational flexibility of a model single-stranded long telomeric sequence. Our findings indicate that its G4 units frequently adopt a beads-on-a-string configuration. We also observe that the interaction between G4 units can be significantly affected by complexation with benchmark ligands. The proposed methodology, which identifies the determinants that govern the formation and structural flexibility of G4 multimers, may be an affordable tool aiding in the selection and design of drugs that target G4s under physiological conditions.

## Introduction

Deciphering the relationship between the structure and function of biological molecules is a difficult but crucial task. This appears even more complex if one considers that in the physiological context the role played by a biomolecule depends not only on the behavior in its monomeric state but also on the interaction among different biomolecules and/or biomolecular units giving rise to higher-order structures. A paradigmatic case where the higher-order structure is crucial is represented by G-quadruplexes (G4s). These biomolecules are noncanonical nucleic acid structures formed by G-rich oligonucleotides which fold into four-stranded helical structures consisting of multiple stacked planar arrays of four guanine bases associated through cyclic Hoogsteen-like hydrogen bonds (G-tetrads).^1^ G4s display three main topologies (parallel, antiparallel, and hybrid) that differ in the relative orientation of the four guanine runs and in the arrangement of loop regions.^2−4^ They are highly polymorphic, as their structure depends both on the specific oligonucleotide sequence and on environmental factors, such as type and concentration of cations, molecular crowding, and/or dehydration conditions.^5−10^ In the genomes of higher eukaryotes, sequences with the ability to form G4s are abundant^11−13^ and concentrated in the telomeric regions (up to 25% of all G4 DNA).^14^ G4s have also been detected in cells,^14,15^ where they are thought to regulate transcription, translation, DNA replication, RNA localization, and other biological functions.^16−18^ Because of such biological importance, G4s have received a lot of attention as drug-design targets.^1,19,20^ In particular, since G4s have been demonstrated to block telomerase and HIV integrase, there is cause to believe that specific G4-stabilizing ligands could be used as anticancer or antiviral drugs.^21,22^ In addition to this, G4s have been widely investigated as promising building blocks and functional elements in fields such as synthetic biology and nanotechnology,^23,24^ mostly because of their high stability, structural versatility, and functional diversity.

The majority of studies on G4s have focused on their monomeric state, but there is evidence that G4s can take many different multimeric forms.^25^ Aggregation has been shown to depend on the length of loops and likely to occur through the stacking of external G-tetrads of parallel folds, with dimers and trimers as the most probable aggregated forms.^9^

Recently, it has been suggested that multimeric G4 structures may play a significant biological role in telomeric DNA. This is due to the ability of the 3′ single-stranded overhang to form higher-order structures consisting of multiple G4 units linked by TTA spacers.^26^ G4 ligands, and in particular anticancer drugs, are known to affect the multimeric state of G4s.^19^ Thus, the successful design of anticancer drugs that target telomere G4s^27^ requires a thorough understanding of the spatial arrangement and stability of their multimeric structure, both with and without potential therapeutic agents.

Very recently, small-angle X-ray scattering (SAXS) experiments have significantly contributed to the representation of the subnanometer details of extended single-stranded human telomere sequences in solution.^26^ The primary challenge lies in extracting all the information from SAXS patterns, which requires the nontrivial combination of ab initio space-filling models^28^ with all-atom molecular dynamics (MD) simulations, as demonstrated by Monsen et al.^26^ In addition, accurately quantifying the strength of base stacking interactions^29−31^ using all-atom MD simulations can be a challenging task, and the large computational demands of these simulations restrict the size of systems that can be investigated and the duration of the explored time-scales.

To provide an expandable, computationally cost-effective, and highly flexible tool for studying the higher-order structural properties of G4s, we propose an approach based on extremely coarse-grained (ECG) simulations. Our approach allows for the direct interpretation of SAXS results from multimers formed by self-assembled Tel22 (d(AGGG(TTAGGG)_3_)) units and the higher-order human telomere sequence Tel72 (d(TTAGGG)_12_) on a case-by-case basis. By using this new method, we provide a quantitative description of stacking energetics and flexibility that are key determinants for the structural properties of G4 multimers. The way the benchmark ligands TMPyP4 porphyrin and BRACO19 are able to promote multimerization is also investigated, since quantifying their effects on stacking energies and topology of G4 multimers is crucial to establish their potential as anticancer or antiviral drugs.

## Results and Discussion

### Self-Assembly of Tel22 Monomers

**Figure 1:**
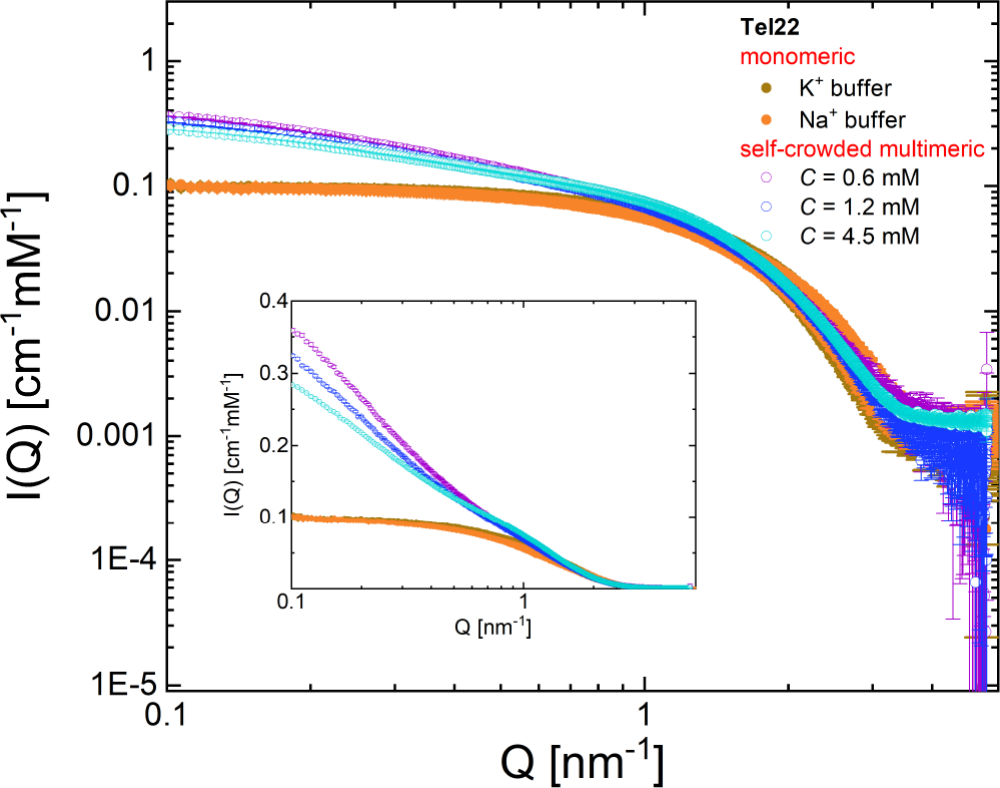
The log–log plot of the SAXS intensities of G4 multimeric samples at different DNA concentrations. Data are reported in absolute scale and normalized to the molar concentration *C* of DNA. For comparison, the SAXS intensities of Tel22 monomeric solutions at *C* = 0.5 mM, prepared in both K^+^ and Na^+^ environments, are shown. In the inset, the same SAXS profiles are shown on a linear–log scale.

DNA multimers composed of self-assembled Tel22 units and standard Tel22 monomers were investigated by SAXS measurements. In Figure 1 we compare the measured intensity of multimeric Tel22 samples, annealed at high DNA concentration and then diluted to 0.6 mM, 1.2 mM, and 4.5 mM, with the signal from the monomer units. It is worth noting that the monomeric samples were prepared at a DNA concentration of *C* = 0.5 mM, in both potassium (K^+^) and sodium (Na^+^) buffer, in such a way as to rule out the presence of possible aggregates (see the Methods section). In the high-*Q* region, *Q* > 1 nm^–1^, where the curves account mainly for the structural features of the Tel22 unit, i.e., shape and characteristic dimensions, through the so-called form factor function *P*(*Q*), the experimental profiles overlap rather well with each other. On the other hand, in the intermediate- and low-*Q* regions, the intensity *I*(*Q*) reflects the higher-order structure arising from the presence of G4 aggregates. Indeed, for *Q* < 1 nm^–1^ their contribution is recognizable from the excess of scattering over the plateau of Tel22 monomers. Size exclusion chromatography (SEC) experiments, performed on the monomeric and multimeric samples, confirm the presence of stable aggregates and a reduced fraction of the monomer state in the latter case (Figure S1).

Interpreting small-angle scattering data is highly challenging because it is inherently difficult to distinguish between the signals of aggregated particles and those of individual molecules. Hence, we exploited numerical simulations using an ECG approach to replicate the current experimental intensities and ascertain the low-resolution structural characteristics of G4 assemblies, where the Tel22 units were modeled as hard cylinders (HCs)^32^ whose dimensions were determined from SAXS experiments. The choice of this cylindrical shape is due to the lack in the PDB database of more detailed structural information on the Tel22 sequence in K^+^ buffer solutions.

**Figure 2:**
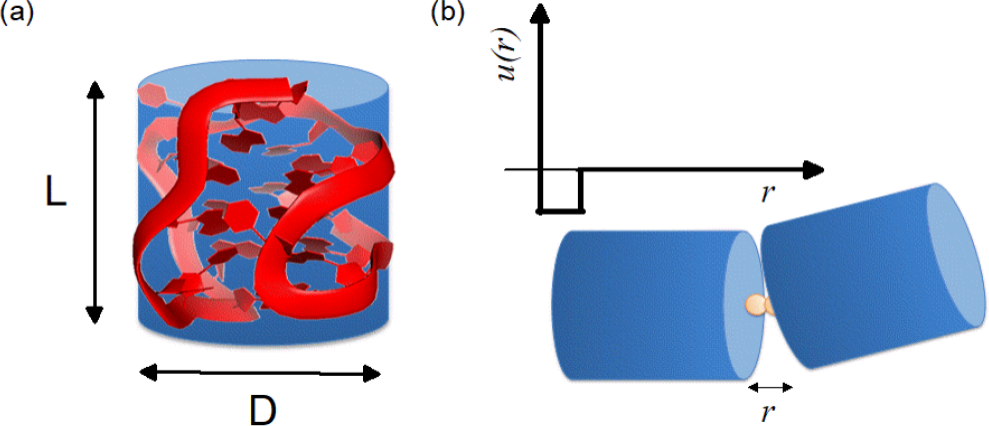
Simulation model for the G4 unit. (a) Each G4 is modeled as a HC characterized by a diameter *D* and a length *L*. (b) Each cylinder is decorated with two attractive sites at the basis. Sites belonging to different cylinders interact through the SW potential *u*(*r*).

In the ECG approach, each HC is decorated with two attractive sites at the basis, as schematized in Figure 2. For more details about this model, see the Methods section. Scattering intensities from MC simulations were obtained following a procedure already successfully used to describe reversible self-aggregation processes in other DNA-based systems and especially suitable for representing the hydrophobic (stacking) forces acting between G4 units.^33−35^

An extensive campaign of simulations was carried out to reproduce the SAXS curves for all the concentrations studied experimentally. In particular, to find the best agreement between simulations and experiments, we explored the parameter space corresponding to different G4 shapes and strength of stacking interaction between Tel22 units. As to the former, the starting point was a HC with a diameter of *D*_0_ = 2.12 nm and a length of *L*_0_ = 3.10 nm that best reproduces the monomeric form of Tel22 sequences (see Figure S2). To modulate the shape of the cylinder, we introduced the parameter *K*, so that *D* = *D*_0_·*K* and *L* = *L*_0_/*K*.

Finally, the stacking interaction between the Tel22 units was varied through the effective temperature T* = *k*_B_*T*/*u*_0_ (where *u*_0_ is the binding energy of the HC attractive sites). ECG simulations can be directly used to obtain quantitative information on stacking and self-assembly of G4s in solution. In particular, the adopted numerical model is consistent with an exponential distribution ν(*l*) of G4 multimer chain lengths *l*: ν(*l*) = ρ*M*^–(*l*+1)^(*M* – 1)^(*l*−1)^, where ρ =  = *N*/*V* is the number of G4s per unit volume, and *M* is the average number of stacked units; see Figure S3). The value of *M* can be directly achieved from the simulation, since it is related to the average potential energy per G4 monomer, ϵ, through the relationship *M* = (1 − ϵ/*u*_0_)^−1^.^36^

**Figure 3:**
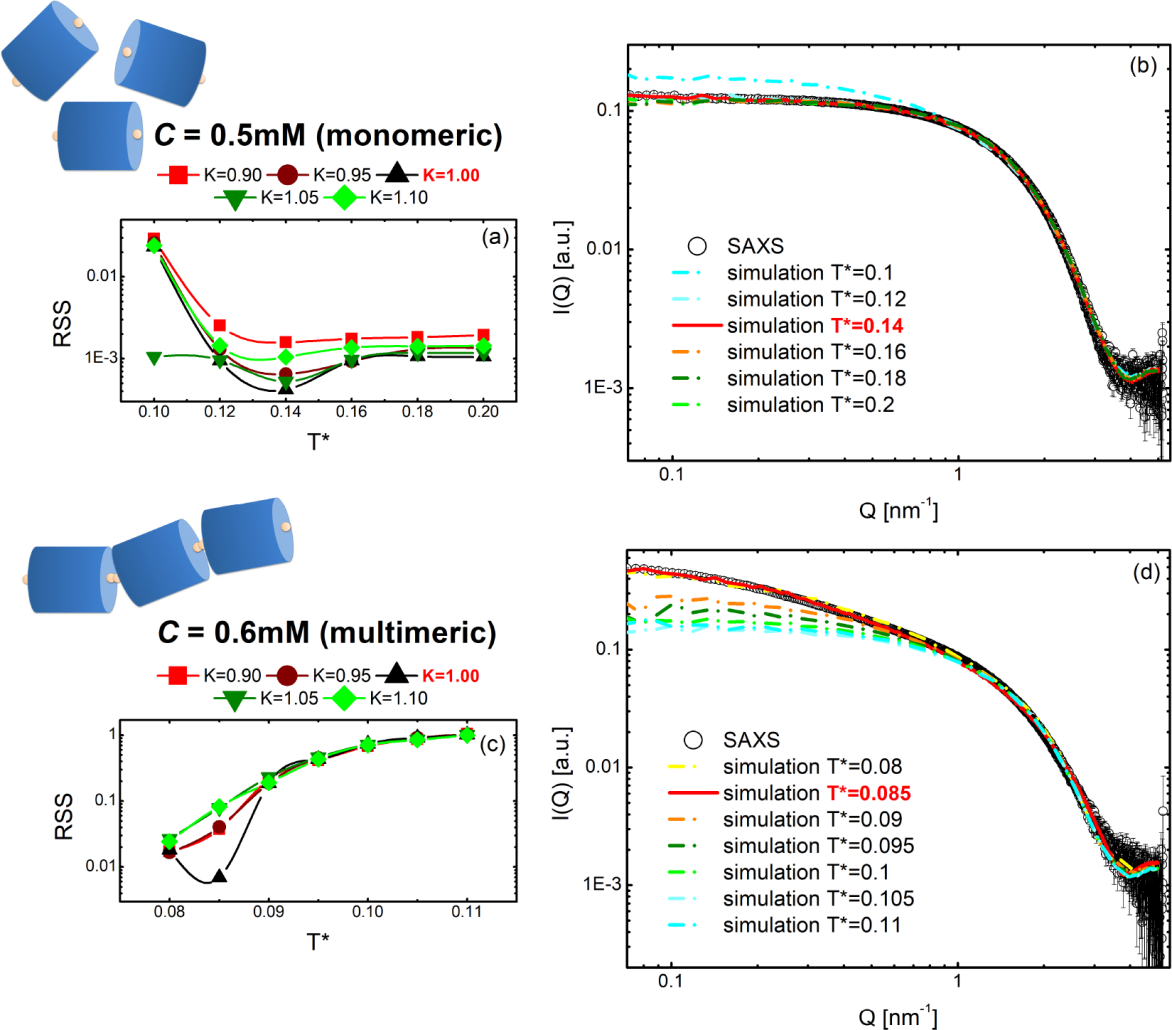
Experimental (symbols) and simulated (lines) scattering intensities for monomeric (panels a and b) and multimeric (panels c and d) G4 samples. Best accordance between experimental and simulated data has been evaluated through the residual sum of squares (RSS), calculated at different values of T* and *K* (panels a and c). At the best value of *K*, the best-temperature simulated curve is reproduced as a solid line.

**Figure 4:**
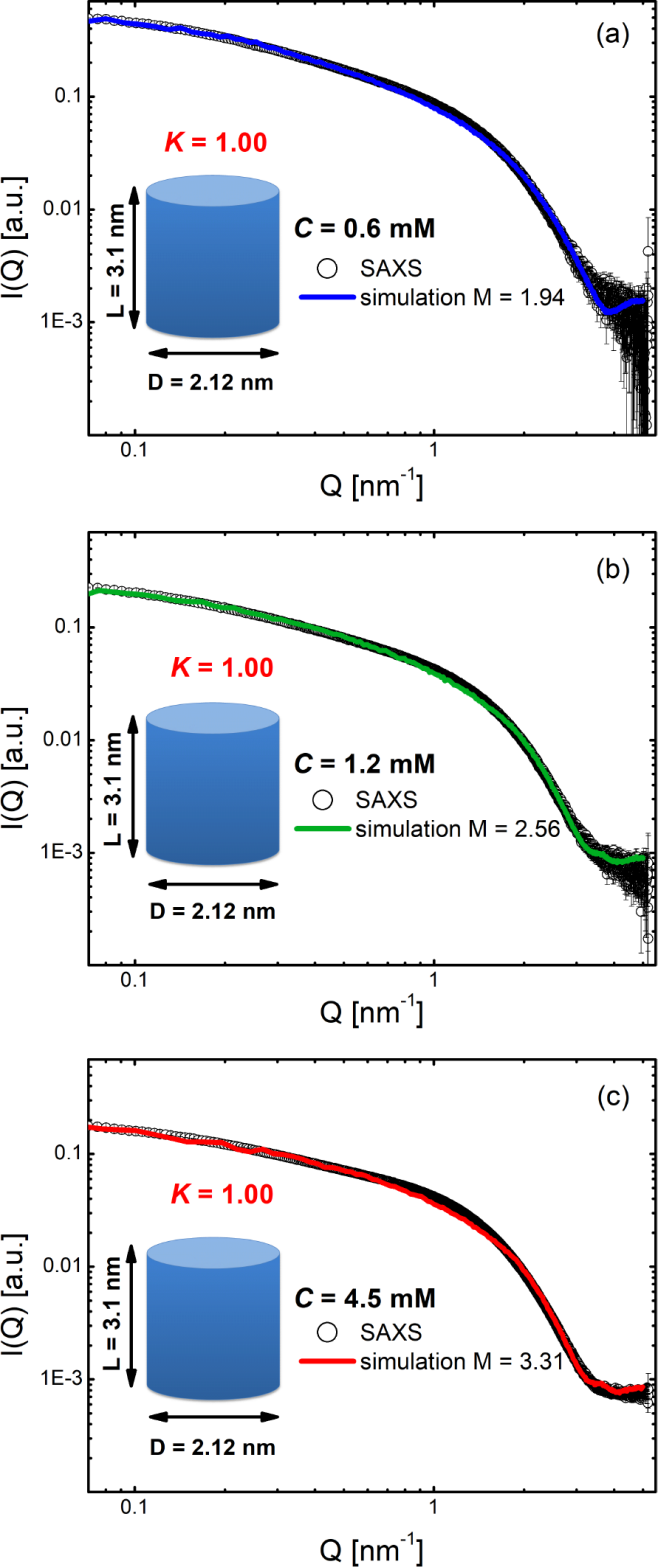
Best accordance between experimental and simulated scattering intensities, respectively, for the G4 solution at *C* = 0.6 mM (a), 1.2 mM (b), and 4.5 mM (c).

The experimental *I*(*Q*) can be suitably reproduced by finely changing the *K* and T* simulation parameters, as shown in Figure 4 where the results from monomeric and multimeric solutions are reported at the indicated concentrations. By checking the modeling on the SAXS profile of Tel22 monomer in K^+^ buffer, the best matching between theoretical and experimental curves was obtained for *K* = 1 and T* = 0.14, corresponding to the condition of noninteracting cylinders (see Figure 3a,b). Indeed, at T* = 0.14 we obtain *M* = 1.02; i.e., only a small percentage of Tel22 units undergo multimerization (see Table 1).

**Table 1:** List of Parameters Associated with the Best Representative State Point (*K*, T*) for Different G4 Samples, Either in Momomeric or Self-Crowding Solutions

monomeric samples	T*	*K*	*M*^a^
Na^+^	0.12	0.95	1.06
K^+^	0.14	1	1.02

					*G*_ST_^0^ b	*H*_ST_^0^ c	*S*_ST_^0^ d
multimeric samples	T*	*K*	*M*	*D*	(kcal mol^–1^)	(kcal mol^–1^)	(cal mol^–1^ K^–1^)
0.6 mM	0.085	1	1.94	1.48	–0.35	–6.85	–22.2
1.2 mM	0.085	1	2.56	1.61	–0.81	–6.85	–20.1
4.5 mM	0.09	1	3.31	1.70	–1.18	–6.47	–18.0

a Average chain length. The error on the values of *M* reported in this and the following table has been estimated to be of the order of 10%.

b Stacking free energy calculated for a standard concentration 1 M of G4s and *T* = 293 K.

c Energy contribution to *G*_ST_^0^.

d Entropic contribution to *G*_ST_^0^.

In addition, the model was found able to capture details from monomers in a different buffer, as shown in Figure S4, where the results for Tel22+Na^+^ and Tel22+K^+^ are reported for comparison. The small difference in the shape-related parameter, *K* = 0.95 for Na^+^, might reflect the different topology in which Tel22 folds in the presence of the two ions.^20,37^ Indeed, Tel22 G4s in Na^+^ solutions take a predominantly antiparallel conformation, which should result in a more elongated structure (lower *K*) than in the K^+^ environment. Also in this case, the propensity to form multimers is very low, in accordance with the obtained value of *M* = 1.06 (see Table 1).

In the case of the self-assembled sample at *C* = 0.6 mM, an excellent agreement between simulation and experiment is obtained for *K* = 1 and T* = 0.085 (Figure 3c,d), with an *M* value of 1.94 confirming the multimeric character of Tel22 aggregates (see Table 1). The complete set of simulations for the three different investigated concentrations can be found in the Supporting Information (Figures S5–S7). Experimental and simulated curves remarkably superimpose onto each other over the whole explored *Q*-range (see Figure 4). In the high-*Q* region, where the SAXS signal is dominated by the form factor, the interpretation of the experimental data deserves great care.^38^ As expected, in this region, the simulated SAXS intensity describes less accurately the experimental data, since the HC geometric unit cannot account for the fine structural details of the actual Tel22 fold. On the other hand, and more interestingly for our goal, simulations are able to reproduce very well the experimental data in the low- and intermediate-*Q* region and can therefore effectively describe the multimerization process.

In particular, the simulations provide an estimate of the average length of G4 multimers at each of the investigated concentrations, as reported in Table 1, where the results for monomers are also shown for completeness. Quite interestingly, *M* values are in the range 1.94–3.31, in agreement with the experimental results suggesting dimers and trimers as the main multimeric forms.^9^ These results are in accordance with those retrieved by fitting the multimer chain length distributions obtained from the simulations with an exponential function (Figure S3). It is worth noting that using an exponential distribution to describe the length of multimer chains is equivalent to assuming that multimerization of G4s complies with a step-growth mechanism. This is analogous to the case of the self-assembly of DNA-encoded nanoparticles into chain-like superstructures^39^ and at variance with the chain-growth mechanism which is less commonly applied to supramolecular biopolymers.^40,41^ In this framework, we can easily estimate the spread of the Tel22 molecular mass distribution as quantified by the ratio between the mass average molecular weight and the number average molecular weight, i.e., the so-called dispersity *D* = 2 – 1/*M*.^42^ The parameter *D* indicates that the self-assembled multimers possess a quite large size distribution, as also confirmed by the SEC measurements, showing the presence of broad peaks associated with multimeric structures.

From the simulations, we find that the HCs in multimers are arranged in coaxial assemblies with an average angle between two adjacent HCs of about 20°, as can be seen in the case of dimers and trimers represented in Figures S8 and S9. This trend is concentration-independent, which is in agreement with the comparable effective temperature values observed in all the samples analyzed. The presence of a well-defined first peak at *Q* = 2 nm^–1^ in the static structure factor *S*(*Q*) calculated from simulations, corresponding to a distance of 3.1 nm between first nearest neighbors, confirms that the HCs within self-assembled multimers are well-stacked (Figure S10).

The knowledge of the average chain length *M* allows us to estimate also the coaxial stacking free energy of the system *G*_ST_ = −*k*_B_*T* ln[*M*(*M* – 1)].^43^ The standard free energy *G*_ST_^0^, calculated for a standard temperature of 293 K and concentration of 1 M, increases from about −0.4 kcal mol^–1^ at the lowest concentration to −1.2 kcal mol^–1^ at *C* = 4.5 mM. The associated contributions of the standard bonding energy *H*_ST_^0^ and the standard binding entropy *S*_ST_^0^ are of the order of approximately −6.5 kcal mol^–1^ and approximately −20 cal mol^–1^ K^–1^, respectively. It is worth noting that the absolute value of *G*_ST_^0^ we calculated is considerably lower than the stacking free energy values reported in the literature for G4 structures. In a recent computational work based on all-atom simulations, for instance, the dimerization free energy of parallel Tel22 G4s was estimated to be of the order of −20 kcal mol^–1^ in the most stable 5′–5′ configuration.^44^ These higher values of stacking energies seem to stem from the nature of the atomistic force fields, which have a tendency of overestimating the stacking free energies.^31^ In fact, a recent study based on all-atom simulations predicted the formation of unrealistically long aggregates for a system consisting of 90 ultrashort DNA duplexes, each comprising 5 base pairs.^31^ In this case, the end-to-end attraction of the short DNA fragments was estimated to be around −6 kcal/mol, a value much larger than the one predicted by the Santa Lucia model (about −1 – −2 kcal/mol)^45^ and from experiments (around −3 kcal/mol).^46^ Since the nature of DNA stacking interactions is very similar to that of attraction between G4 units, our results are consistent with these findings; i.e., our stacking energies for G4s are larger than those estimated for DNA duplexes end-to-stacking,^100^ as expected, but much smaller than those predicted by all-atom simulations. We also observe that more recent versions of atomistic force fields for nucleic acids provide only fine/minor adjustments of the previous force field versions (i.e., changes of the order of few kcal/mol in the interaction energies compared to absolute values of the order of dozens of kcal/mol).^47−49^ In particular, regarding G4 dimerization, there are some indications that the base stacking is often overstabilized in atomistic simulations of nucleic acids with the presently used nonbonded terms.^47^

On these grounds, our coarse-grained approach seems to be able to more effectively calculate the entropic contribution to the stacking free energy, as it happens in the case of self-assembled short DNA-duplexes.^29,30,43,50^ For comparison, computational all-atom MD studies in which only the enthalpic term is considered return G4s stacking energies between −34 and −8 kcal mol^–1^,^51^ which are in line with our values. As current estimates of intermolecular G4 stacking energies mainly come from numerical studies, our findings call for future experimental work on this subject.

As for the shape of the self-assembled cylinders, the ECG simulations provide the same value *K* = 1 for all the investigated systems, thus suggesting that the average G4 unit in multimers has a similar shape as monomeric Tel22 in the presence of K^+^ cation. However, it is worth noting that circular dichroism (CD) experiments reveal that the average topology of multimers significantly changes with increasing concentration, progressively shifting toward the parallel conformation (see Figure S11), in agreement with the literature.^37,52−54^ Quite interestingly, a decomposition of the CD spectra in terms of secondary structural components (Figure S11) providing the contributions from anti–anti, syn–anti, and anti–syn glycosidic angles and from the diagonal and lateral loops^55^ suggests the existence of a possible correlation of the fraction of diagonal and lateral loops with the average number of stacked G4 units *M* (see Figure S12). The decrease in the fraction of diagonal/lateral loops on increasing *M* is consistent with a shift toward a more parallel topology of the Tel22 units upon multimerization.

From the simulations, we can directly estimate the average gyration radius of the G4 multimers, which turns out to be in the range 2.1–3.4 nm (see Figure S13). As a further support of the view provided by our method, the gyration radii are in excellent agreement with those obtained by fitting the SAXS data with a phenomenological function proposed by Beaucage to describe systems with several characteristic lengths.^56^

### Flexibility of High-Order Telomeric G4 Sequences

The Tel22 system is just a small segment of the DNA telomere, which can contain up to 11 copies of G4s in its overhang. The study of long single-stranded sequences d(TTAGGG)_*n*_ in telomeres is a challenging task due to the structural complexity caused by the polymorphism of G4s and their intermediates, such as G-hairpin, G-Triplex, and misfolded long loop. Furthermore, it is still a matter of debate whether higher-order interactions exist between neighboring G4s formed in long telomeric sequences. The question of whether G4s in long telomeric sequences can be described as a flexible ”beads-on-string” structure or whether they form more rigid structures due to stacking interactions remains unresolved.^26,57−60^

**Figure 5:**
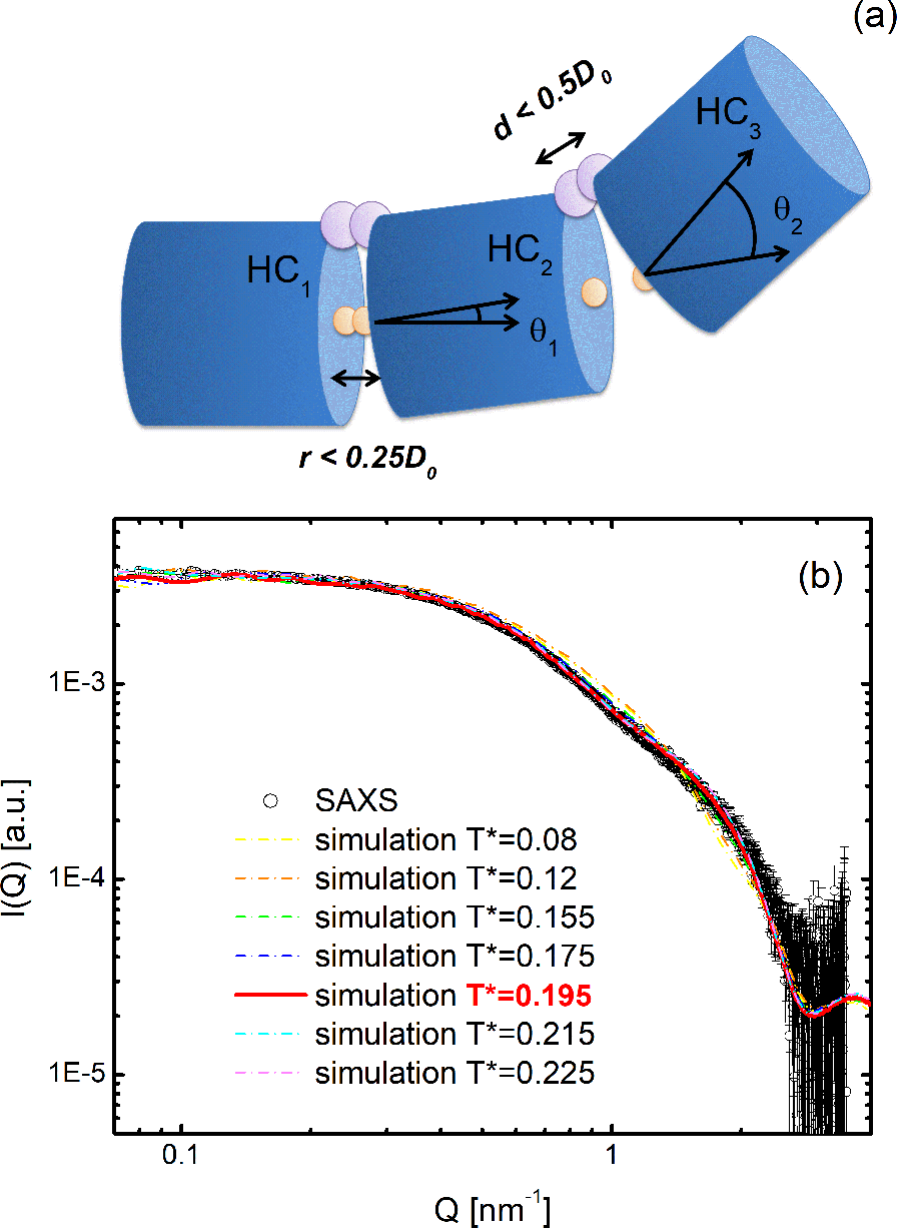
High-order G4 sequences. (a) Each sequence is modeled by three HCs whose shape and stacking interaction (yellow patch) are identical to those used in the previous sections. An additional covalent interaction (light purple patch) has been employed, as described in the Methods section. (b) Experimental (symbols) and simulated (lines) scattering intensities for Tel72. At the best value of *K*, the best-temperature simulated curve is reported as a solid red line.

**Figure 6:**
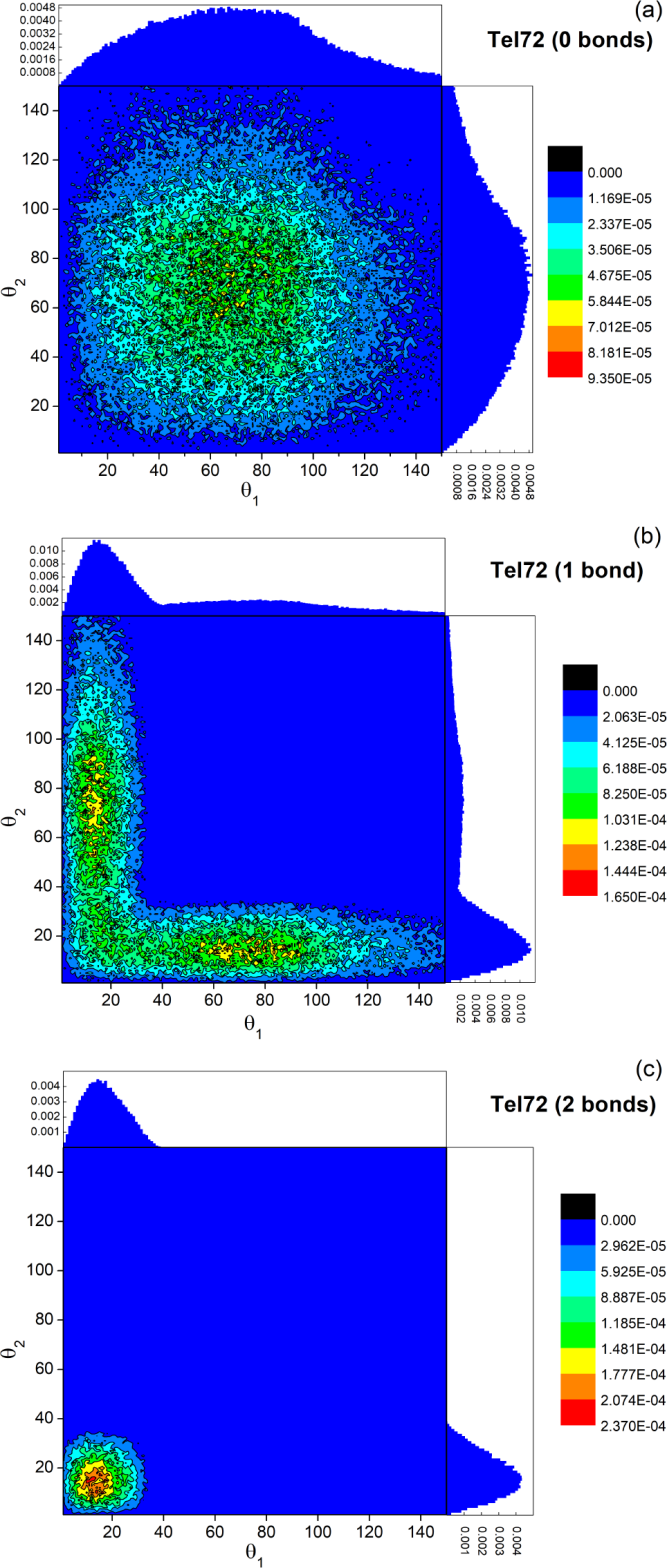
Distributions of angles θ_1_ and θ_2_ formed by two adjacent cylinders within the trimer, as obtained from the best-fit simulation. The distributions corresponding to (a) 0 bonds, (b) 1 bond, and (c) 2 bonds between the HCs are reported.

To shed light on this subject, we examined the SAXS pattern of the sequence Tel72 from previous experiments^26^ deposited on the online database SASBDB.^61^ Unlike the self-assembly of Tel22 units, a G4 trimer was modeled with our ECG approach as being composed of three HCs. In addition to the attractive sites placed in the center of their bases to mimic stacking interactions, the HCs also contain additional interaction sites located on the edge of their bases to account for the linkers between G4 units (Figure 5a). To reproduce the SAXS experimental curve, we explored the phase space of the parameters T* and *K*, similarly to the case of self-assembled multimers. In Figure 5b we show that the data are excellently modeled by the ECG simulations for values of T* = 0.195 and *K* = 1.4. To describe the flexibility of the Tel72 sequence, we analyzed the distribution of angles θ_1_ and θ_2_, which are formed by the axes of HC_1_ and HC_2_ and by HC_2_ and HC_3_, respectively (Figure 5a). These angles correspond to the cases where 0, 1, and 2 bonds are formed between the HCs in the trimer. In the case where 0 bonds form, shown in Figure 6a, the 2D angular distribution is symmetric and centered at about θ_1_ = θ_2_ = 69°. Quite interestingly, 45% of the trimers populate this distribution. Different is the case when the G4 units form 2 bonds, where the distribution is again symmetric and centered around the values θ_1_ = θ_2_ = 15° (Figure 6c) and only accounts for 10% of the trimers. It is worth noting that this estimate of totally stacked G4 trimers is quite lower compared to the value of about 38% recently obtained by applying a molecular dynamics based analysis on the same set of SAXS data.^26^ Finally, when just one bond is formed, an angular distribution with features intermediate between those obtained for 0 and 2 bonds (see Figure 6b) describes the remaining 45% of trimers. Overall, our findings support a picture where a significant number of trimers are in a “beads-on-a-string” arrangement, consistent with the rather low value we found for the stacking energy, i.e., high value for T*.

### Sampling Ligand-Induced Multimerization

As a further validation of our integrative method and in order to test other routes of aggregation, ECG simulations were applied to Tel22 G4s complexed with two benchmark ligands, namely, TMPyP4 and BRACO19, which are able to induce G4 multimerization.^62^ The former is a cationic porphyrin that offers great promise due to its favorable stacking to G-quartets in terms of molecular size, planarity, positive charges, and hydrophobicity, and the latter is a trisubstituted acridine compound, developed as a ligand for stabilizing G4 structures and representing one of the most potent cell-free inhibitors of human telomerase.^63,64^ Both of the ligands were investigated in Tel22 K^+^ solutions. Also in this case, a set of several simulated data were optimized on the SAXS curves of the Tel22+TMPyP4 and Tel22+BRACO19 solutions. Due to the rather small contribution to the molecular volume from the drug, an *effective* HC which accounts for G4 complexed with ligands was considered in the simulations.

**Figure 7:**
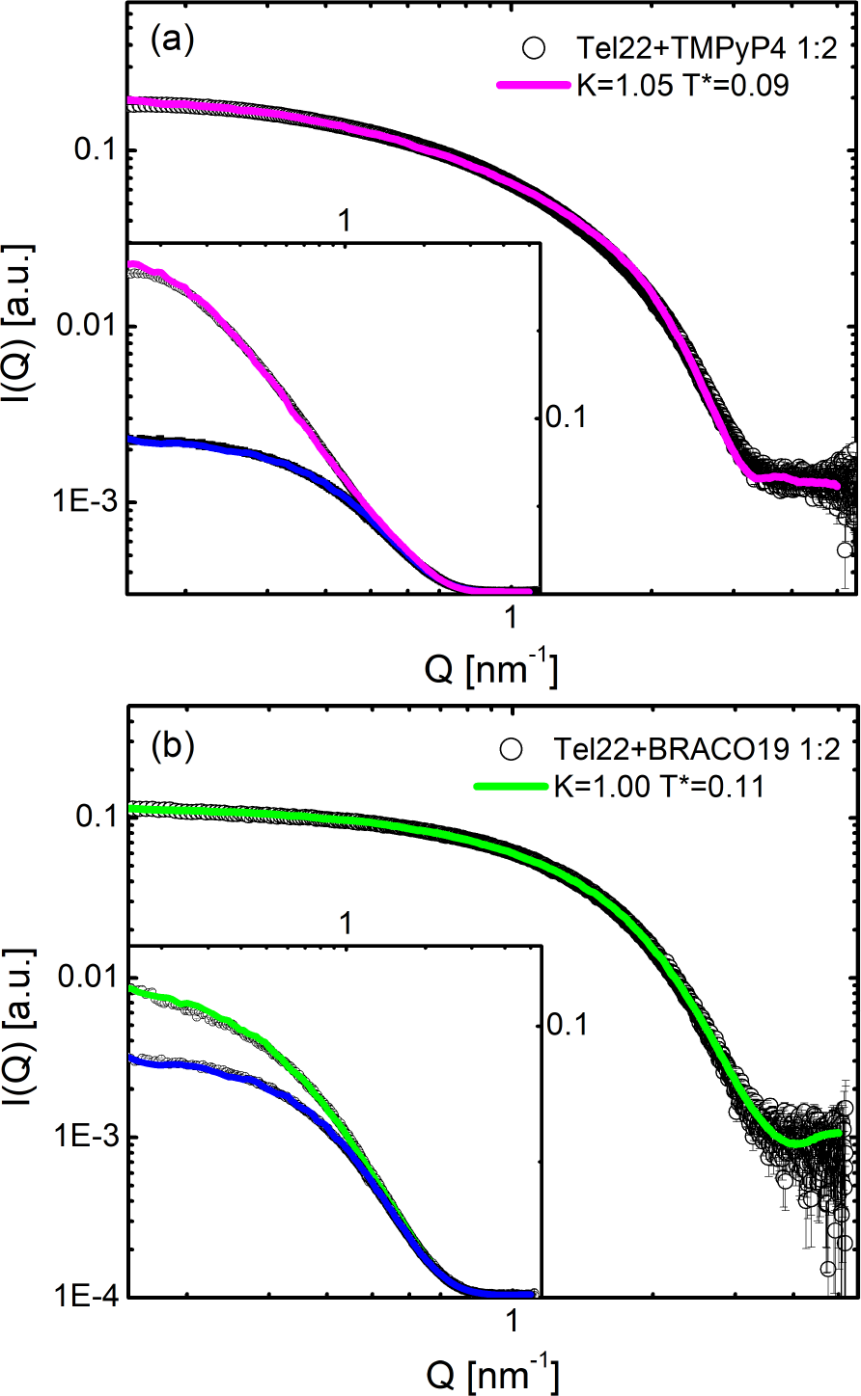
Experimental and simulated scattering intensities for Tel22+TMPyP4 (a) and Tel22+BRACO19 (b) solutions. Insets show in the semilogarithmic logscale the same curves together with the monomer profiles (blue lines).

The best match between the theoretical and experimental profiles was achieved with the values reported in Table 2. We found that TMPyP4 is able to promote aggregation of Tel22 G4s better than BRACO19 (*M* = 1.66 vs *M* = 1.13), as confirmed from the fact that the low-*Q* curve of Tel22+TMPyP4 deviates from the monomeric one much more than Tel22+BRACO19 (see the insets of Figure 7). This is probably due to both the peculiar shape and the size of the porphyrin molecule, which provide high matching with the G4 scaffolds and favors stacking between monomers. This supports quite recent results where a mixture of monomers and dimers was found to be suitable to reproduce SAXS data for Tel22+TMPyP4.^32^ On the other hand, at least at the investigated molar ratio, BRACO19 only slightly shifts the monomer/multimer thermodynamic equilibrium, likely because it can establish binding modes also with G4 loops,^65,66^ thus making less probable the stacking of Tel22 units. It is worth noting that the present method has the potential to be used also to obtain structural details at the level of the G-tetrad element, once further experimental techniques with higher spatial resolution are employed. This kind of upgrade would allow us to directly investigate also the case of ligands intercalating between two G-tetrads.^67^

**Table 2:** List of Parameters Associated with the Best Representative State Point (*K*, T*) for Samples of G4s in the Presence of Ligands

					*G*_ST_^0^	*H*_ST_^0^	*S*_ST_^0^
sample	T*	*K*	*M*	*D*	(kcal mol^–1^)	(kcal mol^–1^)	(cal mol^–1^ K^–1^)
TMPyP4	0.09	1.05	1.66	1.40	–0.05	–6.47	–21.9
BRACO19	0.11	1	1.13	1.12	1.12	–5.29	–21.9

## Conclusions

In this work, we show how by combining experimental (SAXS) and computational (ECG simulations) low-resolution structural techniques, it is possible to provide a quantitative description of telomeric G4 multimers as promoted by self-assembly or composing long single-stranded sequences. By using our integrated approach, we are able to describe a challenging system consisting of polydisperse aggregates of polymorphic G4s while also determining their structural flexibility. Extensive exploration of the wide range of parameters related to the strength of the stacking interaction between G4 units and their shape was carried out using ECG simulations. The simulations were refined to successfully reproduce the experimental SAXS data. As the DNA concentration increases, the average length and stability of G4 self-assembled multimers increase, with the aggregates assuming a coaxial disposition. It is noteworthy that the detailed information we have obtained would be challenging to acquire through the use of analytical models that account for a system of polydisperse multimers of interacting G4s to describe the SAXS patterns.

In the case of the G4 units within the trimers formed by the physiologically relevant long telomeric sequence Tel72, only a small fraction of them correspond to fully stacked configurations. This results in a spatial arrangement that is reminiscent of a beads-on-a-string system.

Our approach offers a description of the large-scale structure and stability properties of transient G4 multimers, which can provide valuable information on the presence of available binding sites for drugs between adjacent G4 units. Additionally, it elucidates how specific ligands promote or hinder interunit stacking interactions that may be relevant for biological functionality. On these grounds, our method can complement information from other high-resolution experimental/numerical techniques able to investigate multimers formed by multiple DNA/RNA strands.^68−70^ Due to their versatility, ECG simulations can achieve an optimal match with experiments even for highly complex systems of G4 multimers. Also the presence of different coexisting types of G4s could be accounted for. The populations of these structures can be derived from the comparison of ECG simulations with experimental data, and the corresponding thermodynamic distribution can be estimated accordingly. Based on these findings, we propose that ECG simulations are a valuable computational tool that can be used in conjunction with all-atom MD simulations. The latter can be utilized to design and model new ligands, while the former can be more effective in testing these ligands for systems that are closer to physiological conditions.

## Methods

### Experimental Methods

#### Sample Preparation

Human telomere Tel22 AG_3_(T_2_AG_3_)_3_ (*M*_*w*_: 6966.6 g mol^–1^) was purchased from Eurogentec and used as received. Samples were prepared by dissolving the lyophilized powder in a 50 mM phosphate buffer at pH 7, 0.3 mM EDTA, and 150 mM KCl. The high concentration of the starting solution (≈13 mM) is appropriate to ensure the formation of aggregates, which remain stable even after dilution. The presence of multimers in analogous samples of the telomeric sequence G_3_(T_2_AG_3_)_3_ has been revealed by PAGE by Palacky et al.,^54^ especially in samples annealed at the highest K^+^ and DNA concentrations, as in the case of the present investigation. The solution was heated up to 95 °C, slowly cooled to room temperature, and left at room temperature overnight. After this procedure, the solution was centrifuged for 120 s at 15 °C and 15 000 rpm. From the centrifuged solution, samples at 3 different DNA concentrations were prepared, namely, *C* = 0.6 mM, 1.2 mM, and 4.5 mM. The molarity of the solutions was determined from UV absorption measurements at 260 nm, using a molar extinction coefficient of 228 500 M^–1^ cm^–1^. Both experimental and computational investigations were performed at these concentrations. Before measurements, samples were further annealed and left at room temperature overnight. Conversely, the monomeric state samples were prepared from a stock solution with a lower concentration of *C* = 1 mM. Such a different procedure avoided the formation of aggregates. As for the Tel22 sample in the Na^+^ environment, the lyophilized powder was dissolved in 100 mM sodium phosphate at pH 7.4. Concerning the Tel22-ligand solutions, the DNA was prepared using the same procedure as for the G4 monomers. After the annealing it was complexed with TMPyP4 and BRACO19 in the 1:2 [DNA]/[ligand] stoichiometric molar ratio, corresponding to 0.5 mM Tel22 and 1 mM ligand.

#### Small-Angle X-ray Scattering

Small-angle X-ray scattering (SAXS) experiments were performed at the BM29 beamline of the European Synchrotron Radiation Facility (ESRF) in Grenoble, France. The incident energy was 12.5 keV, corresponding to an incident wavelength of 0.99 Å^–1^. The scattering vector range was between *Q* = 0.0044 Å^–1^ and 0.521 Å^–1^. All patterns were collected at 20 °C. Analogous patterns of the buffer were collected before and after every collection on the samples and used to subtract any contribution from the solvent and the sample environment.

### Computational Methods

#### Simulation Models

##### Monomers

The simulation model consisted of hard cylinders (HCs) characterized by a length, *L*, and a diameter, *D*, with two reversible attractive sites at the bases. The attractive sites were located along the symmetry axis at a distance *L*/2 + 0.15*D*/2 from the HC center of mass. Sites belonging to distinct particles interact via the square-well (SW) potential, i.e., β*u*_SW_ = −β*u*_0_, if *r* < δ, and β*u*_SW_ = 0, if *r* > δ, where *r* is the distance between the interacting sites, δ = 0.5 nm is the interaction range (i.e., the diameter of the attractive sites), and β*u*_0_ is the ratio between the binding energy and the thermal energy *k*_B_*T*, where *k*_B_ is the Boltzmann constant. The temperature was expressed as the adimensional parameter T* = *k*_B_*T*/*u*_0_. To calculate the structure factor corresponding to an ensemble of interacting cylinders with homogeneous scattering length, each cylinder was replaced with a set of scattering points randomly placed inside its volume with a fixed number density.^35^ This method ensures that the numerical scattering intensity also includes the form factor of HCs so that it can be directly compared with the experimental one. It is noteworthy that in this model the dimensions of the hard cylinder effectively account for the hydration shell.

##### Trimers

G4 trimers were modeled by three HCs, whose shapes and reversible attractive interactions are identical to those used to model G4 monomers and that are held together by covalent interactions. The latter ones have been accounted for through interactions sites placed on the edge of HCs bases as shown in Figure 5a. Note that upper and lower terminal HCs do not have reversible interaction sites on their external bases, since we assume that trimers do not attract each other. Covalent sites belonging to two adjacent cylinders in the trimers interact through a potential such that it is infinite if their distance *d* is greater than 0.5*D*_0_, or 0 otherwise. The interaction between trimers has been modeled via the SW potential as in the monomer case, using a value of δ = 0.25*D*_0_ = 0.53 nm. Scattering patterns for the trimers were calculated by employing the same procedures discussed above for monomers.

#### Monte Carlo Simulations

Simulations were performed in the canonical (NVT*) ensemble, leveraging a recently developed algorithm for checking the overlap between HCs,^71^ which relies on a novel and very efficient algorithm for finding the roots of a quartic equation.^72^

##### Monomers

For each value of concentration, we simulated a suitable number of particles *N* in a cubic box with volume *V* using standard periodic boundary conditions. Values of *N* and *V* were chosen so that the density ρ = *N*/*V* = *N*_av_*C*, where *N*_av_ is Avogadro’s number, reproduced each of the experimentally investigated values of DNA concentration *C*. The number of particles used in the simulations was *N* = 6292 for all of the investigated concentrations. The aspect ratio of the HCs was varied from that associated with the starting values of *D*_0_ = 2.12 nm and *L*_0_ = 3.1 nm by introducing the parameters *K*: *D* = *D*_0_·*K* and *L* = *L*_0_/*K*. For each value of *C*, five different values of *K* (*K* = 0.9, 0.95, 1, 1.05, 1.1, 1.15, and 1.2) were considered, thereby obtaining a total of 21 starting configurations. Each starting configuration was thermalized for at least 10^6^ MC steps at six different values of T* ranging between T* = 0.08 and 0.2. The initial configuration used for the equilibration was obtained by placing the hard cylinders on an orthorhombic lattice.

##### Trimers

For this model we simulated *N* = 6000 HCs, i.e., 2000 trimers, at a density corresponding to a concentration of 10 mg/mL. The aspect ratio *K* of the HCs was varied from 0.80 to 1.80, and for each *K* we carried out simulations over reduced temperatures ranging from 0.08 to 0.225. Starting from an initial configuration where HCs were placed on an orthorhombic lattice, we thermalized the system for at least 2 × 10^5^ MC steps. Latter values ensure, for all cases studied, that potential energy attains a stationary value.

## Supporting Information Available

Size exclusion chromatography experiments; form factor of the Tel22 monomer; multimer chain length distribution; comparison between G4 monomers in different buffer solutions; comparison between experimental and simulated intensities for high-concentration samples; angular distribution of self-assembled dimers and trimers; structure factor; circular dichroism of high-concentrated samples; comparison between different estimates of the average dimension of G4 multimers (PDF)
