# Supplementary material for: Stacking Interactions and Flexibility of Human Telomeric Multimers
Source: J Am Chem Soc. 2023 Jul 11;145(29):16166–75. doi: 10.1021/jacs.3c04810 (PMC10375521; doi:10.1021/jacs.3c04810)
Supplement: Supplementary file 1 — ja3c04810_si_001.pdf [file ja3c04810_si_001.pdf]

Supporting Information for: Stacking interactions and Flexibility of Human Telomeric Multimers

Benedetta Petra Rosi,[†,#] Valeria Libera,[†,‡] Luca Bertini,[†] Andrea Orecchini,[†,‡] Silvia Corezzi,[†] Giorgio Schirò,[¶] Petra Pernot,[§] Ralf Biehl,[‖] Caterina Petrillo,[†] Lucia Comez,[*,‡] Cristiano De Michele,[*,⊥] and Alessandro Paciaroni[*,†]

†*Department of Physics and Geology, University of Perugia, via Alessandro Pascoli, 06123 Perugia, Italy*

‡*CNR-IOM, Department of Physics and Geology, University of Perugia, via Alessandro Pascoli, 06123 Perugia, Italy*

¶*CNRS, Institut de Biologie Structurale, 71 avenue des Martyrs, 38044 Grenoble, France*

§*European Synchrotron Radiation Facility (ESRF), 71 avenue des Martyrs, 38043 Grenoble, France*

‖*Jülich Centre for Neutron Science and Institute of Biological Information Processing (JCNS-1/IBI-8), Forschungszentrum Jülich GmbH, 52425 Jülich, Germany*

⊥*Department of Physics, University of Rome La Sapienza, piazzale Aldo Moro 2, 00185 Rome, Italy*

#*Current address: Jülich Centre for Neutron Science and Institute of Biological Information Processing (JCNS-1/IBI-8), Forschungszentrum Jülich GmbH, 52425 Jülich, Germany*

E-mail: comez@iom.cnr.it; cristiano.demichele@uniroma1.it; alessandro.paciaroni@unipg.it

1. Size exclusion chromatography experiments

Size exclusion chromatography (SEC) experiments were performed with an AZURA Fast Protein Liquid Chromatography system (Knauer) using a Superdex 200 Increase 10/300 GL column. The column was equilibrated with K^+ buffer (same used for the sample preparation for CD and SAXS). Samples were eluted at a flow rate of 0.9 mL/min.

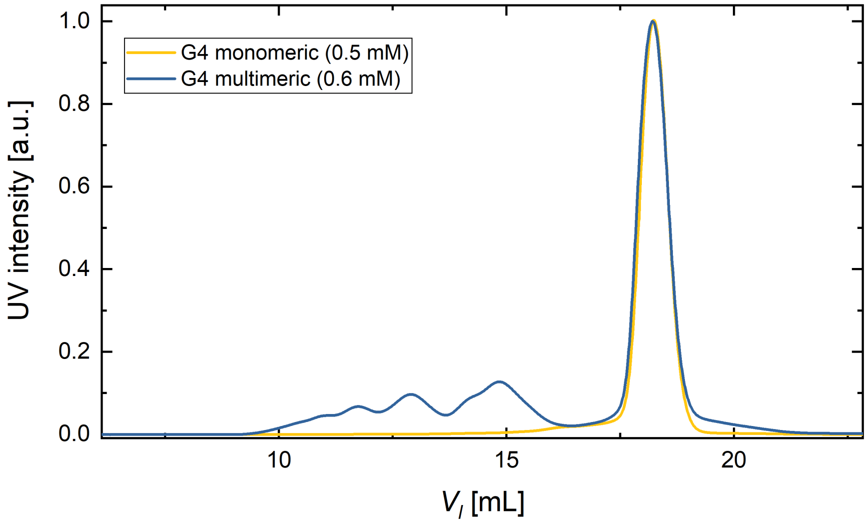

Figure S1. Chromatograms of G4 samples obtained with different annealing protocols and concentrations, as a function of the elution volume V_l.

Although SEC is unable to predict the molecular weights of quadruplex scaffolds (and chain-length distribution), since no quadruplex calibration standards exist, it can be used to separate quadruplex monomeric and multimeric species and to evaluate their relative content in a sample, revealing changes in equilibrium due to e.g. annealing conditions.[1] Figure S1 shows the comparison between the elution traces of G4 samples at similar DNA concentration but obtained by using different annealing protocols. It is apparent that both samples exhibit a prominent peak at an elution volume of $V_l \approx 18$ mL, which is likely attributable to G4 monomers. In the multimeric sample, additional peaks are present at smaller elution volumes. The peaks are well separated from each other, indicating stable multimeric species. To attain more quantitative information, we determined the percentage

*The authors L.C., C. D. M., and A. P. equally contributed to the manuscript.

of monomers in the multimeric sample by calculating the ratio of the area under the monomer peak to the total area of the size exclusion chromatography (SEC) trace. The calculated value ($\sim$ 60%) is in good agreement with that obtained from simulations by using the exponential law distribution (52%).

2. Form factor of the Tel22 monomer

The SAXS profile of the Tel22 monomeric solution is well reproduced by a cylinder form factor. By fitting the experimental data through the SasView package (https://www.sasview.org/), we obtained for the diameter and the length values equal to D_0=2.12 $\pm$0.02 nm and L_0=3.10 $\pm$0.05 nm, respectively (see Figure S2).

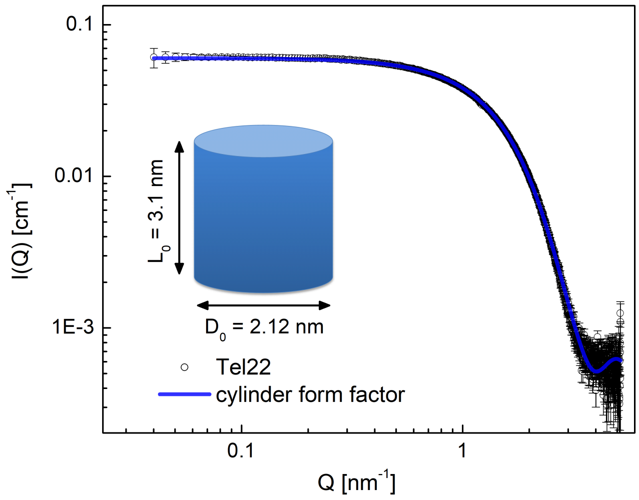

Figure S2. SAXS pattern of Tel22 sample (C=0.5 mM) in K^+ buffer solution (circle). The form factor was reproduced using a cylindrical shape by means of the SasView Software Package (blue solid line).

3. Multimer chain length distribution

From ECG simulations we derived the distribution of multimer chain lengths for all the investigated concentrations. The corresponding results are reported in Figure S3 along with the best fit using the normalized exponential distribution:

$$\nu_N(l) = (M-1)^{l-1}/M^l \quad \text{(S1)}$$

The obtained values of M are 1.92±0.01, 2.49±0.02 and 3.42±0.04 for C = 0.6 mM, 1.2 mM and 4.5 mM, respectively.

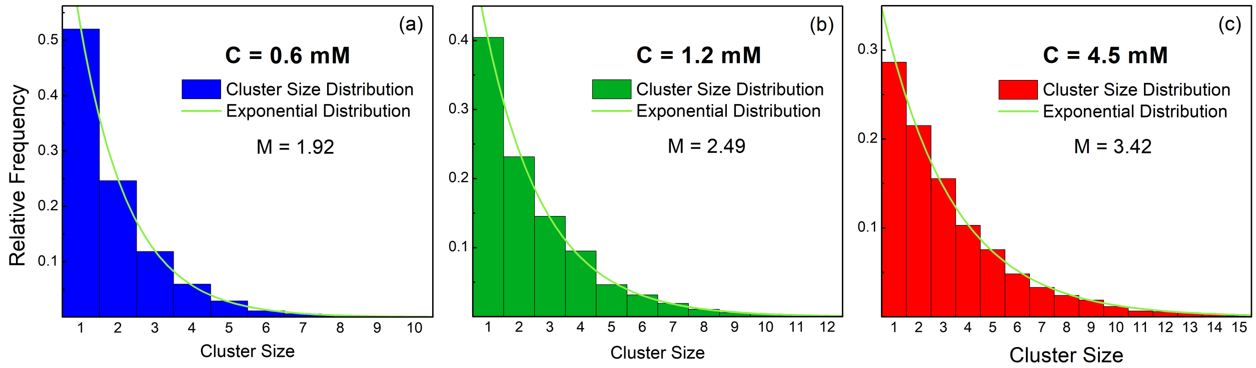

Figure S3. Cluster Size Distribution for the sample at (panel a) C = 0.6 mM, (panel b) C = 1.2 mM and (panel c) C = 4.5 mM, along with the best fits using Equation S1.

4. Comparison between G4 monomers in different buffer solutions

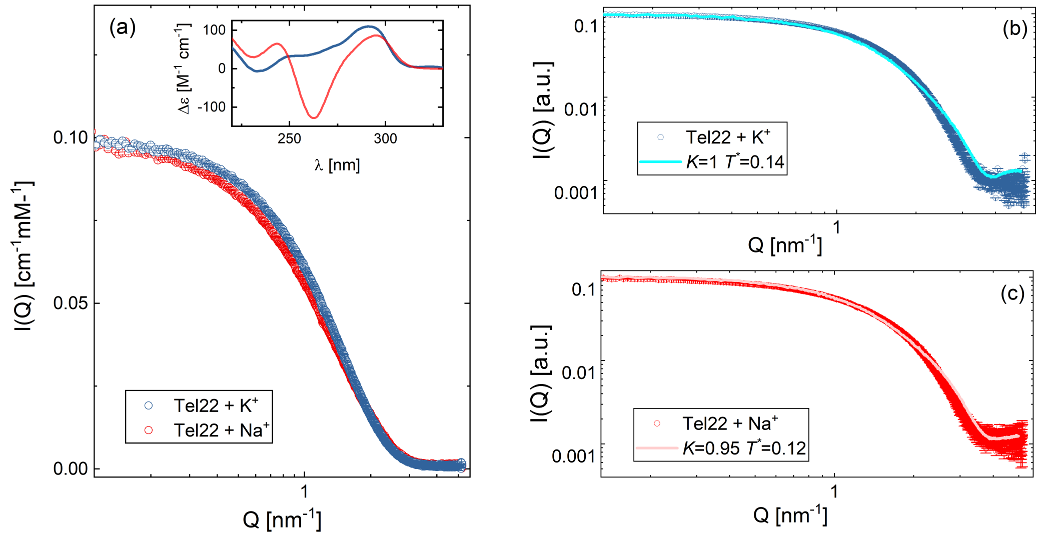

Figure S4. Panel a: SAXS curves of G4 samples (C=0.5 mM) in K^+ (blue) and Na^+ (red) buffer solutions. In the inset, the CD spectra of the same samples are reported. Panel b and c show the best agreement between the experimental SAXS data and the simulated intensities respectively for the K^+ and Na^+ sample.

Tel22 monomeric solutions were measured by SAXS and CD techniques in both K^+ and Na^+ buffer at the same C=0.5 mM DNA concentration (Figure S4 a). CD profiles show the hybrid-like and antiparallel-like signatures assumed by Tel22 respectively in K^+ and Na^+

environments. Figures S4 b and c display that ECG simulations were able to reproduce the main low-resolution features of the two different structures.

5. Comparison between experimental and simulated intensities for high-concentration samples

ECG simulations were performed using a thick grid of T^* and K values to interpret the SAXS measurements. In Figures S5, S6 and S7, the comparison between experimental and simulated scattering intensities for samples with DNA concentration respectively of $C = 0.6$ mM, 1.2 mM and 4.5 mM is reported. For $C = 0.6$ mM, the best accordance is found when the simulation parameters assume the values of $K = 1$ and $T^* = 0.085$; for $C = 1.2$ mM, the best matching is for $K = 1$ and $T^* = 0.085$, and finally for $C = 4.5$ mM, the corresponding best parameters are $K = 1$ and $T^* = 0.09$.

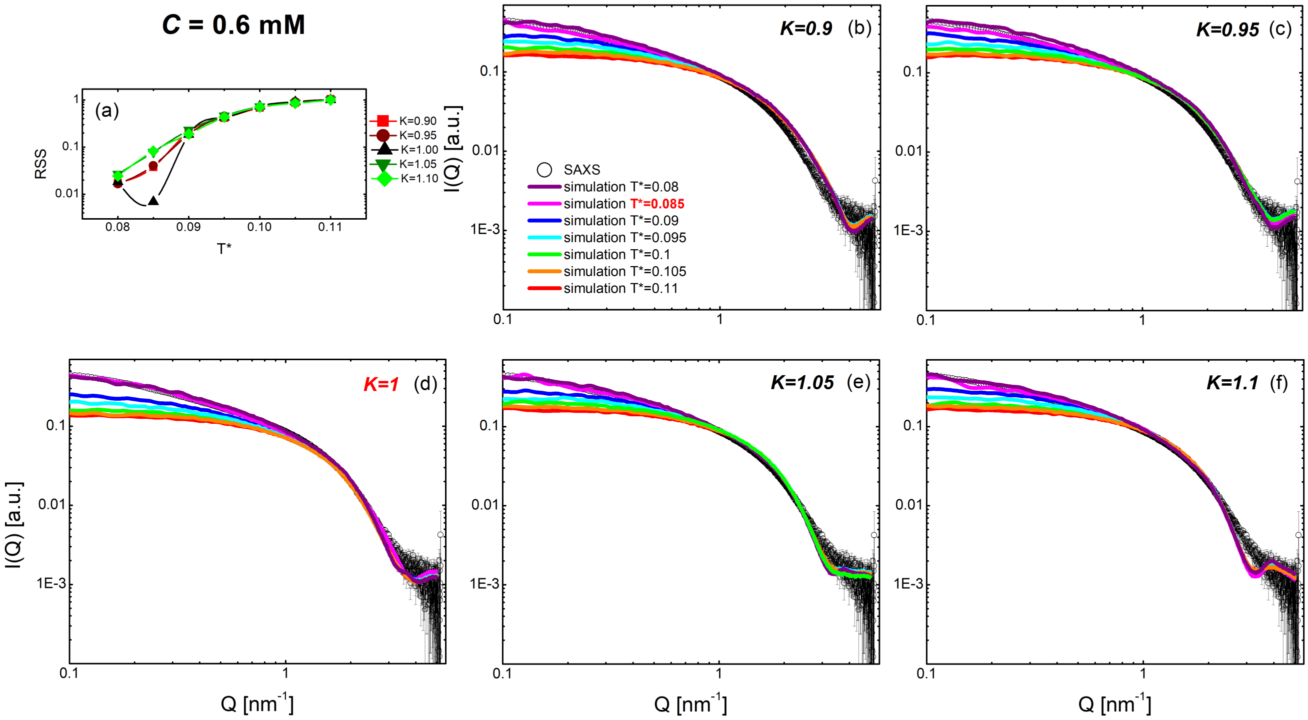

Figure S5: Scattering intensities for the quadruplex solution at $C = 0.6$ mM, obtained from SAXS measurements and simulations (panels b – f). The accordance between experimental and simulated data has been evaluated through the residual sum of squares (RSS), calculated at different values of T^* and K (panel a).

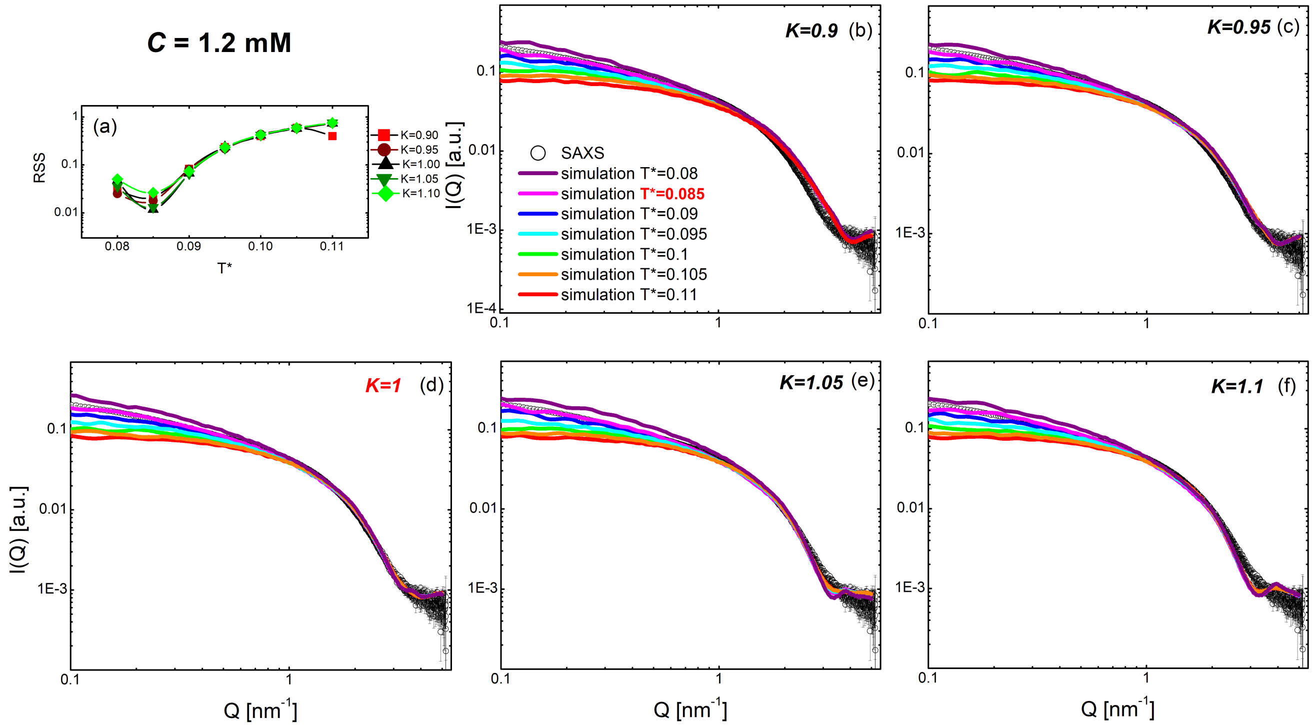

Figure S6: Scattering intensities for the quadruplex solution at $C = 1.2$ mM, obtained from SAXS measurements and simulations (panels b − f). The accordance between experimental and simulated data has been evaluated through the residual sum of squares (RSS), calculated at different values of T^* and K (panel a).

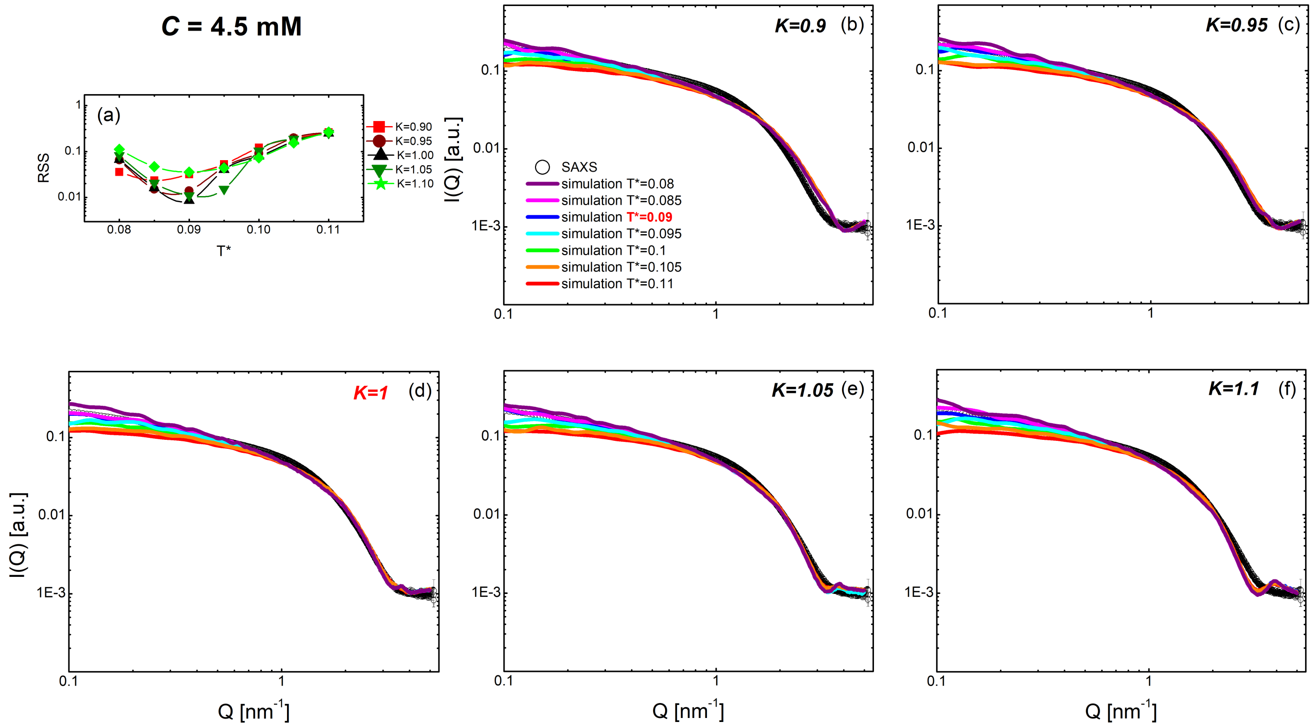

Figure S7: Scattering intensities for the quadruplex solution at $C = 4.5$ mM, obtained from SAXS measurements and simulations (panels b − f). The accordance between experimental and simulated data has been evaluated through the residual sum of squares (RSS), calculated at different values of T^* and K (panel a).

6. Angular distribution of self-assembled dimers and trimers

In order to investigate the structural flexibility of the self-assembled G4 multimers, we derived from the ECG simulations the distributions of the angles formed by adjacent hard cylinders within dimers (Figure S8) and trimers (Figure S9) for all the investigated concentrations. All the distributions are centered around $\theta_0 \simeq 20$, suggesting that multimers preferentially stack into a coaxial arrangement. This trend is found to be concentration-independent, in accordance with the similar values of T^* obtained for the best-fit simulations.

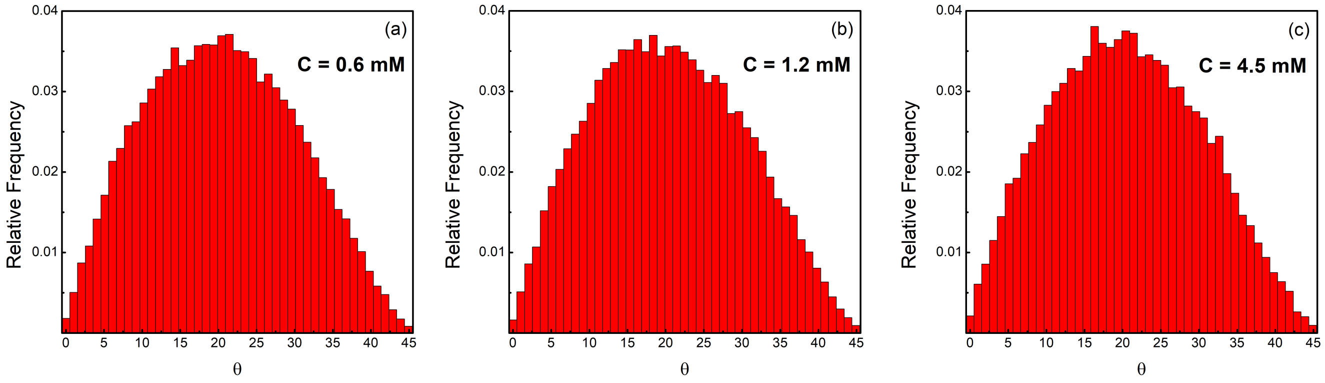

Figure S8: Distribution of the angle formed by two G4 units in a self-assembled dimer, respectively for the G4 solution at $C = 0.6$ mM (panel a), 1.2 mM (panel b), and 4.5 mM (panel c).

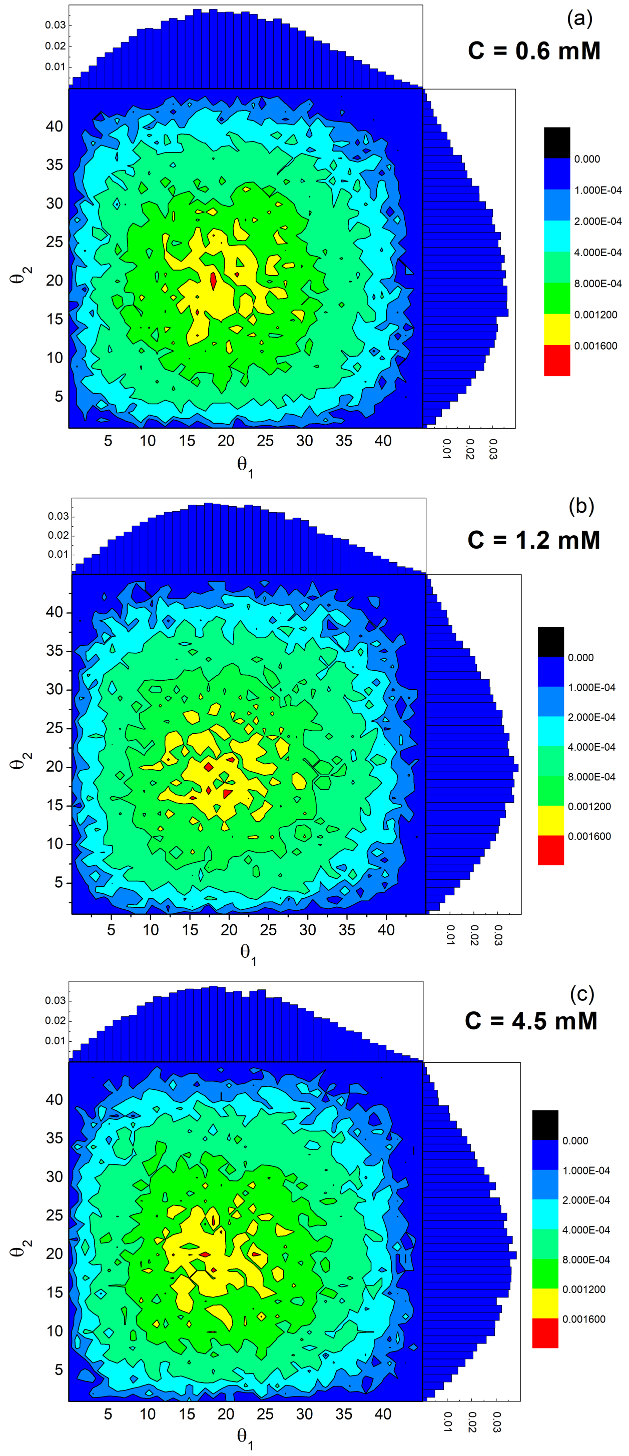

Figure S9: Distributions of the angles θ_1 and θ_2 formed by two adjacent cylinders within the trimer as obtained from the best-fit simulation. The distributions corresponding to: (panel a) 0 bonds, (panel b) 1 bond and (panel c) 2 bonds between the HCs are reported.

7. Structure factor

The static structure factor $S(Q)$ was calculated from the ECG simulations according to the procedure described in *Pal et al.*[2] The resulting profiles are reported in Figure S10. We notice that the value of $S(Q)$ in the low-Q region decreases with increasing the concentration. Such a behaviour reveals how the structure factor affects in a non trivial way the low-Q signal of the measured SAXS intensities.

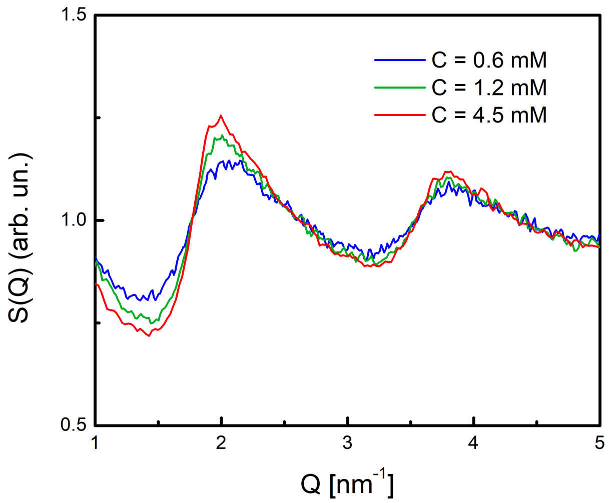

Figure S10: Structure factor obtained from the simulation for $C = 0.6$ mM (blue line), 1.2 mM (green line), and 4.5 mM (red line).

8. Circular dichroism of high-concentrated samples

Circular dichroism (CD) experiments on Tel22 samples at concentrations of 0.6 mM, 1.2 mM, and 4.5 mM were performed using a Jasco J810 spectropolarimeter in 0.01 to 0.1 mm path-length quartz cells. Spectra were collected in the range from 220 nm to 330 nm with a scan speed of 50 nm/min. All the CD profiles were acquired at room temperature. CD data were expressed as the difference in the molar absorption $\Delta\epsilon$ [M^{-1} cm^{-1}] of the right- and left-handed circularly polarized light.

As shown in Figure S11, at $C = 0.6$ mM (panel a) the spectrum displays the typical features of G4 hybrid topology, with a maximum at about 295 nm, a shoulder at 270 nm and a

minimum at 240 nm. As C increases, the maximum progressively downshifts and a new peak appears at 265 nm (panel b and c), suggesting an increase in the parallel fraction consistent with previous literature.[3] A method based on the principal component analysis and singular value decomposition was used to quantify the fraction of the major G4 topologies.[4] In Figure S11 the fit of experimental data obtained from the deconvolution into the three main folded topologies (parallel, antiparallel and hybrid), is reported, with the corresponding results represented in the pie charts. A population transfer is evident, as the hybrid component halves at the highest concentration, while the parallel component grows from 10% to 40%. In addition, in order to quantify the secondary structural element steps mainly involved in the multimerization process, a decomposition of the CD spectra is reported in terms of the glycosidic angle of the base steps, associated with anti–anti, syn–anti or anti–syn conformations, in diagonal or lateral loops, or in other sub-unities (see Figure S11, panel d-f). Based on these results, we propose that the secondary structure components associated with the syn-anti and diagonal/lateral loop segments are correlated with the average number of G4 units in the multimer M obtained from ECG simulations (see Figure S12). This suggests a potential secondary/quaternary structural connection through multimerization.

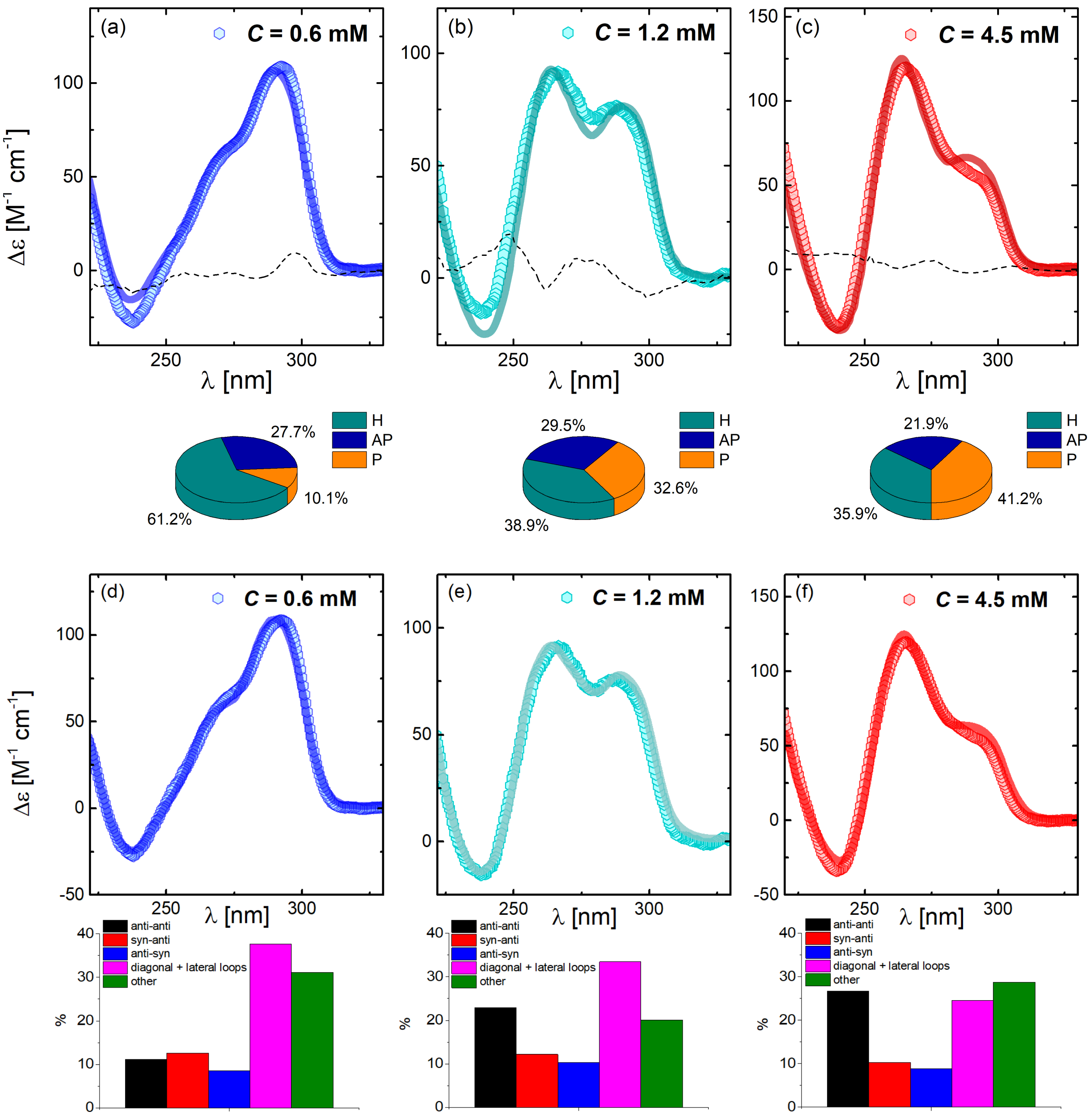

Figure S11: CD spectra of G4 solutions at different DNA concentrations (circles). In each panel, the fit resulting from the spectral deconvolution in terms of tertiary strcuture (panels a-c) through the software by del Villar-Guerra *et al.* is reported (solid lines), together with the corresponding residues (dashed lines). Pie charts in the bottom of each panel report the fraction of quadruplex units with antiparallel (AP), hybrid (H), and parallel (P) conformation, according to the spectral deconvolution. The Tel22 secondary structure is also given in terms of the glycosidic angle of the base steps, associated with anti–anti, syn–anti or anti–syn conformations, in diagonal or lateral loops, or in other sub-unities. The corresponding fit curves are shown in the panels d-f. The hystograms at the bottom report the percentages of each species at the indicated concentrations.

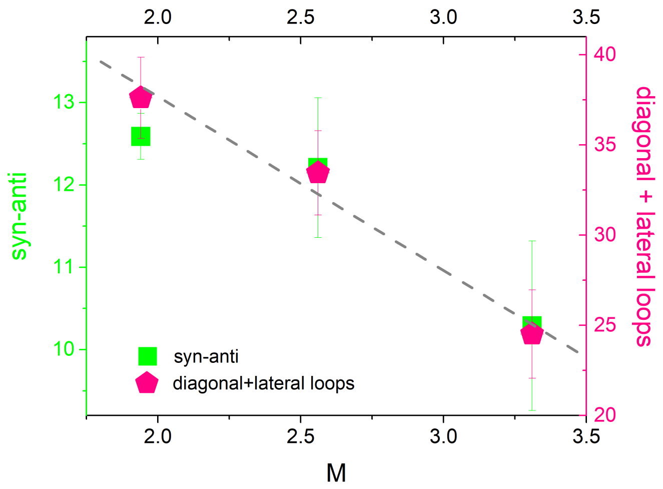

Figure S12: Percentage of the syn-anti and diagonal/ lateral loops contributions plotted versus the parameter M, which is the average number of stacked units determined from ECG simulations.

9. Comparison between different estimates of the average dimension of G4 multimers

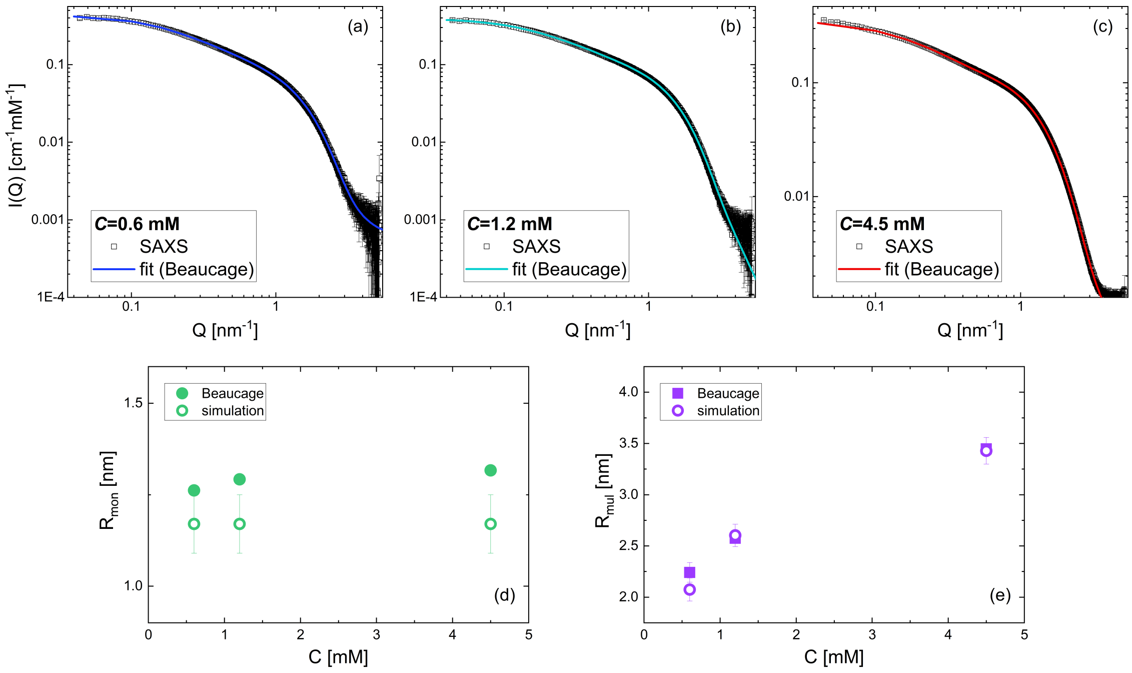

Figure S13: Panel a, b and c report the comparison between SAXS data and the corresponding fitting function according to Equation S2, at concentration $C = 0.6$ mM, 1.2 mM and 4.5 mM respectively. Values of the radius of gyration of the G4 monomer (R_{mon}) and of the G4 multimers (R_{mul}) are compared to the corresponding values obtained by the simulations in panel d and e respectively.

An estimate of the average size of G4 multimers can be obtained by fitting the experimental data using global scattering functions able to describe the behaviour of polymer chains and other structural systems with multiple structural levels:[5,6]

$$\begin{aligned} I(Q) \simeq\ & A_1 \exp(-Q^2 R_{agg}^2/3) + A_2 \exp(-Q^2 R_{mul}^2/3)(1/Q^*)^{P_{mul}} \\ & + A_3 \exp(-Q^2 R_{mon}^2/3) + A_4 (1/Q^*_{mon})^{P_{mon}} + bck, \end{aligned} \tag{S2}$$

where $Q^* = Q/[\,\mathrm{erf}(QkR_{agg}/6^{1/2})]^3$ and $Q^*_{mon} = Q/[\,\mathrm{erf}(Qk_{mon}R_{mon}/6^{1/2})]^3$, A_i $(i = 1, 4)$ are constant amplitudes and bck gives the constant background. The first term in Equation S2 describes the very low Q region and takes into account large-scale scattering objects (aggregates of G4 multimers) with size R_{agg}. The second term describes the mass-scaling regime at intermediate Q with two structural limits, which are given by the small-scale size R_{mon} (related to the dimension of the monomers) and the intermediate-scale size R_{mul} (related to the dimension of small multimers). The last two terms describe the behaviour of the quadruplex structural unit at high Q. The fitting parameters for the three concentrations are reported in Table SI1. The intensity curves are well described by a Porod behaviour for the G4 monomer ($P = 4$), whereas the multimers approach the fractal behaviour of a polymer in good solvent conditions ($P_{mul} \approx 1.5$).[6]

Table SI1: Parameters obtained by fitting the SAXS data by means of the expression reported in Equation 1.

C [mM]	R_{agg} [nm]	R_{mul} [nm]	R_m [nm]	P^a	P_{mon}	k^b	k_{mon}
0.6	10.5 ± 0.2	2.24 ± 0.09	1.26 ± 0.01				
1.2	11.8 ± 0.03	2.58 ± 0.03	1.29 ± 0.01	1.33 ± 0.04	4	1.06	1.06
4.5	13.6 ± 0.04	3.45 ± 0.02	1.32 ± 0.01				

[a] The value was obtained by fitting the low-concentration data and kept constant for the other two values of C. [b] Values of k and k_{mon} where chosen according to Reference.[6]

In Figure S13, the experimental data together with the corresponding fitting functions are reported. It is interesting to compare the values of R_{mon} and R_{mul} obtained by fitting the SAXS data with those evaluated by the simulations. Specifically, R_{mul} was directly

retrieved from the simulations, while $R_{mon} = L^2/12 + R^2/2$, where L and $R \equiv D/2$ are the characteristic dimensions of the HC unit. From Figure S13(d) and (e), it can be observed how there is a good agreement between experiments and simulations.

References

(1) Miller, M. C.; Trent, J. O. Resolution of Quadruplex Polymorphism by Size-Exclusion Chromatography. *Current Protocols in Nucleic Acid Chemistry* **2011**, *45*, 17.3.1–17.3.18.

(2) Pal, A.; De Filippo, C. A.; Ito, T.; Kamal, M. A.; Petukhov, A. V.; De Michele, C.; Schurtenberger, P. Shape Matters in Magnetic-Field-Assisted Assembly of Prolate Colloids. *ACS Nano* **2022**, *16*, 2558–2568.

(3) Renčiuk, D.; Kejnovská, I.; Školáková, P.; Bednářová, K.; Motlová, J.; Vorlíčková, M. Arrangements of human telomere DNA quadruplex in physiologically relevant K^+ solutions. *Nucleic Acids Res.* **2009**, *37*, 6625–6634.

(4) del Villar-Guerra, R.; Trent, J. O.; Chaires, J. B. G-Quadruplex Secondary Structure Obtained from Circular Dichroism Spectroscopy. *Angew. Chem. Int. Ed.* **2018**, *57*, 7171–7175.

(5) Beaucage, G.; Kammler, H. K.; Pratsinis, S. E. Particle size distributions from small-angle scattering using global scattering functions. *J. Appl. Crystall.* **2004**, *37*, 523–535.

(6) Beaucage, G. Small-Angle Scattering from Polymeric Mass Fractals of Arbitrary Mass-Fractal Dimension. *J. Appl. Crystallog.* **1996**, *29*, 134–146.
